# Implication of fibroblast growth factor 7 in ovarian cancer metastases and patient survival

**DOI:** 10.3389/fonc.2024.1524606

**Published:** 2025-01-16

**Authors:** Laura F. Mortan, Brooke A. Meelheim, Justin Garland, Jacqueline A. Bohn, Zitha Redempta Isingizwe, Doris M. Benbrook

**Affiliations:** ^1^ Gynecologic Oncology Section, Stephenson Cancer Center, Obstetrics and Gynecology Department, University of Oklahoma Health Sciences Center, Oklahoma City, OK, United States; ^2^ Pathology Department, University of Oklahoma Health Sciences Center, Oklahoma City, OK, United States

**Keywords:** cancer, metastases, ascites, fibroblast growth factor 7, FGF7, survival

## Abstract

**Background/Objectives:**

Patients with ovarian cancer commonly experience metastases and recurrences, which contribute to high mortality. Our objective was to better understand ovarian cancer metastasis and identify candidate biomarkers and drug targets for predicting and preventing ovarian cancer recurrence.

**Methods:**

Transcripts of 770 cancer-associated genes were compared in cells collected from ascitic fluid versus resected tumors of an ES-2 orthotopic ovarian cancer mouse model. Associated cell types and pathways were explored with bioinformatics. FGF7 protein was measured using capillary-based immunoassays or ELISA in mouse and clinical specimens. Significances of differential gene expression and patient prognosis were determined by volcano plot and log-rank test, respectively.

**Results:**

Tumor transcriptomes exhibited higher endothelial cells, oxygenation, proteasome activity, and metabolism in comparison to ascites, but similar percentages of cancer-associated fibroblasts and immune cells. FGF7 mRNA was significantly higher in mouse tumors compared to ascites. FGF7 protein was significantly higher in tumors than in ascites in independent mouse models and clinical specimens. Serum FGF7 protein levels above the median of 25 patients with ovarian cancer were associated with worse progression-free and overall survival (p = 0.005 and 0.019, respectively) independent of patient and tumor characteristics.

**Conclusions:**

In comparison to ascites, tumors exhibit different transcriptomic profiles that identify candidate biomarkers and drug targets for predicting and preventing recurrence. Among these, elevated tumoral FGF7 validated at the protein level and elevated serum FGF7 were significantly associated with worse patient survival. These results support further development of FGF7 receptor-targeted drugs and serum FGF7 to prevent and predict recurrence, respectively.

## 1 Introduction

Epithelial ovarian cancer is a highly lethal malignancy due to its common diagnosis at late-stage disease where it has already spread beyond the fallopian tubes and ovaries. After primary treatment and control with a platinum- and taxane-based chemotherapy combination, ovarian cancer commonly recurs. Recurrences that occur more than 6 months following initial treatment typically respond to retreatment with platinum-based chemotherapy**;** however, the cancers often recur with decreasing time frames in between each recurrence (1). Patients with platinum-resistant disease (recurrence <6 months from the last platinum therapy) have more limited options and a survival estimate of <17 months (2, 3). Recent advances in ovarian cancer maintenance therapy have dramatically improved the lives of patients. Inhibition of angiogenesis with bevacizumab and/or poly (ADP) ribose polymerase (PARP) with olaparib, niraparib, or rucaparib has proven effective in prolonging disease-free survival for ovarian cancer patients (4–6). However, these maintenance therapies are limited by the development of resistance and toxicities (7, 8).

Ovarian cancer metastases and recurrences primarily disseminate through the peritoneum, where accumulation of ascitic fluid is a common occurrence with increasing frequency with more advanced cancer stages. The ascitic fluids of ovarian cancer patients exhibit a diverse composition comprising a mixture of normal, cancer and immune cells, spheroids of cells, and acellular components (9, 10). The tumor microenvironment in ascitic fluid has been shown to promote tumor metastasis, immune evasion, and chemoresistance (9, 11).

Multiple approaches have been used to identify new targets for developing strategies to treat patients with ovarian cancer. The specific targets that have been evaluated fall into categories of proteins, microRNAs, long non-coding RNAs including circular RNAs, biomarkers of stem cells, epithelial-to-mesenchymal transition or DNA repair, DNA mutations or methylation patterns, histone acetylation patterns, and extrachromosomal circular DNAs (12). Because ovarian cancer is highly heterogeneous and complex, bioinformatic approaches have been applied to DNA mutation, gene or protein expression, or metabolomic profiling of blood, tumor tissue, and/or ascites specimens from patients with ovarian cancer in efforts to better understand how ovarian cancer develops, progresses, recurs and becomes resistant to treatment (13–16). An important element of these approaches is to validate differential expression of specific proteins using multiple experimental models, clinical specimens, and experimental techniques.

The primary objective of this project was to identify and validate a protein differentially expressed in ovarian cancer cells present in solid tumors compared to ascites, which could be targeted in the development of a strategy to prevent the establishment of tumors from ascitic fluid in patients with ovarian cancer. We observed that the mRNA expression of *fibroblast growth factor 7 (FGF7)* was detected at significantly higher levels in cells of the attached tumor, compared to the ascites, of an orthotopic model of ovarian cancer using human ES-2 ovarian cancer cell line in immunodeficient mice. Higher expression of FGF7 in tumor compared to ascites cells was validated at the protein level in two replicates of the orthotopic model and in an independent immunocompetent mouse model using the syngeneic murine ID8 *Trp53* ***^−^*** *^/^* ***^−^*** luc cell line. Worse patient survival was associated with higher levels of tumoral FGF7 mRNA in public databases and serum FGF7 protein in our independent set of clinically annotated specimens.

## 2 Methods

### 2.1 Cell lines and chemicals

ES-2/GFP-luc (hereafter called ES-2) ovarian cancer cell line (17) (gifted by Dr. Branimir I. Sikic, Standford University, Stanford, CA, USA) was authenticated by the short tandem repeat method and proven to be negative for mycoplasma, Hantaan, and lymphocytic choriomeningitis viruses prior to use. Paclitaxel (Cat. # 33069-62-4, LKT Laboratories, St. Paul, MN, USA) was dissolved in a 1:1 ratio of ethanol to Cremophor EL (Cat. # HY-Y1890, MedChem Express, Monmouth Junction, NJ, USA).

### 2.2 Discovery and replicate orthotopic mouse models of ovarian cancer

Eight female athymic nude Fox1nu mice (Cat. # 6903F, Envigo, Indianapolis, IN, USA) were injected through the intraperitoneal (i.p.) route with one million ES-2 cells in 100 µl of phosphate-buffered saline. After the injection, the animals were monitored and weighed daily and their tumors imaged twice per week. To image the tumors, mice were sedated with isoflurane, and then XenoLight D-Luciferin K+ Salt Bioluminescent Substrate (Cat. # 122799, PerkinElmer, Waltham, MA, USA) was injected i.p. into the mice at a concentration of 150 mg/kg per mouse. After 10 to 15 min, the luminescent signals in the mice were imaged and measured using an *In Vivo* Imaging System (IVIS). After 15 days of treatment, mice were euthanized, and their ascites fluid was collected. Then, invasive solid tumors inside the peritoneum were surgically resected, combined, weighed, and frozen in liquid nitrogen. Cells in the ascites fluid were collected through centrifugation, placed in a cryovial, which was then submerged in liquid nitrogen to cryopreserve the specimens. The specimens were stored at −80°C until use as described below. A replicate of this model was conducted as described for the discovery model, with the exception that 10 million ES-2 cells were injected. No treatment groups were included in this second model.

### 2.3 Independent validation orthotopic mouse model of ovarian cancer

A modified syngeneic mouse ID-8 *Trp53^−/−^* cell line (provided by Iain A. McNeish, University of Glasgow) was used. The ID-8 *Trp53^−/−^* cell line has a heterozygous p53 null mutation and produces aggressively growing tumors of high-grade serous ovarian cancer (HGSOC) histology when injected into the peritoneum of C57Bl/6 mice (18). In contrast, the parental ID-8 cell line produces slow-growing tumors that lack the characteristic mutation profiles of HGSOC (19). Luciferase-expressing ID-8 *Trp53^−/−^* cells were generated by transduction of ID-8 *Trp53^−/−^* with firefly luciferase-expressing lentivirus (BPS Bioscience 79692-H) and selected with hygromycin (200 µg/ml) until all cells in the vehicle control cultures were eliminated. Luciferase expression was validated using Promega Luciferase Assay System (Promega, E1500). The ID-8 *Trp53^−/−^* luc cells were grown to 80% confluence in 2-D cultures and, then, were plated on an ultra-low-attachment 96-well plate (50,000 cells/well) and incubated overnight to form spheroids. All spheroids from each 96-well plate were pooled and injected into the peritoneal cavity of 6- to 8-week-old female C57BL/6NHsd mice using a 21-gauge needle (N = 13–14 per group, Envigo). The mice were i.p. injected with saline starting on the same day as the spheroid injection and then daily throughout the experiment. Tumor size was measured weekly by IVIS as described above. Total body weight and abdomen circumference were used to monitor overall health and ascites formation. When humane endpoints were reached, the experiment was terminated, and specimens were collected as described for the ES-2 model above.

### 2.4 RNA or protein isolation

RNA or protein was isolated by placing cell pellets from ascites specimens or 50 mg of cryopreserved tumor into 200 µl of DNA/RNA Shield (Cat # R1200-25, Zymo Research) or 200 µl of T-PER (Cat. #PI78510, ThermoFisher, Waltham, MA, USA), respectively, and homogenized in a 1.5-ml tube using a Bullet Blender Navy Bead Lysis Kit (Cat. # NAVYE1-RNA, Next Advance, Troy, NY, USA). RNA quality and concentration were determined using the Eukaryote Total RNA Pico Series II assay run on the Bioanalyzer 2100 (Agilent Technologies, Santa Clara, CA, USA). Only RNA samples that had RNA Integrity Numbers greater than 5 were used. Protein concentrations were measured using the BCA Protein Assay Kit (Cat. # 23225, Pierce Biotechnology, Waltham, MA, USA).

### 2.5 NanoString analysis

RNA that was isolated from tumor and ascites specimens and passed the quality control testing described above was evaluated for bulk expression analysis using the NanoString Human Signaling Tumor Signaling 360 panel (Cat. NS_HS_TUMORSIG_v1.0, NanoString Technologies, Seattle, WA, USA). This panel consists of 770 genes involved in tumorigenesis, metastasis, and inflammation. Using NSolver, individual genes that exhibited raw data counts below 0.5 fM expression in the positive control and ≤2 standard deviations above the mean of the negative controls were eliminated from the analysis. One matching set of ascites and tumor specimens was omitted from the analysis because the raw data generated with these specimens did not meet these quality control criteria. Next, the raw data of the samples were corrected for background by subtracting the geometric mean counts of 20 housekeeping. A threshold count of 10 was used to categorize genes as being below the level of detection in the analysis. The normalized data were then exported for bioinformatic analysis.

### 2.6 Bioinformatic analysis

Data were analyzed using the ROSALIND online software (https://rosalind.bio/), with a HyperScale architecture developed by ROSALIND, Inc. (San Diego, CA). The limma R library was used to calculate fold changes and p-values and perform optional covariate correction (20). Using the fpc R library, which takes into consideration the direction, type of all signals on a pathway, position, role, and type of every gene, the partitioning around medoids (PAM) method was used for clustering and generation of the final heat map of the differentially expressed genes (21). The parameters of the analysis were set to have significance if the Log2 of the fold change was −1.5 or 1.5 and had a Q value less than or equal to 0.05. Also, Ingenuity Pathway Analysis (IPA, Qiagen, Hilden, Germany) was performed to identify pathways associated with the differentially expressed gene patterns and determine if they were elevated or reduced in the tumor compared to those in the ascites.

### 2.7 Deconvolution analysis

Raw counts from the NanoString analysis were used in R 4.3.1 and assessed with the package immunedeconv. Deconvolution analysis was done using the MCPCounter method to compare samples, cell types, or both. The MCPCounter method allows between- and within-sample comparisons and generates a score in arbitrary units as described (22, 23). Percentages of cell types were predicted using the used gene expression data and the ESTIMATE algorithm in R.4.3.1. as described (24).

### 2.8 Gene Ontology pathway analysis

GO enrichment analysis was performed in R4.3.1 and included genes that had p values less than 0.05 and fold differences in expression between a Log2 of −1.5 to 1.5. The reference package used to identify GO pathways was org.Hs.eg.db, which provides genome-wide annotations of the human genome. Gene symbols from the dataset were mapped to this reference package, and associated pathways were generated using the clusterProfiler package gseGO function. The top 20 pathways were selected based on the level of significance and overall suppression or activation of the pathway.

### 2.9 FGF7 enzyme-linked immunosorbent assay

Equal serum volumes or protein lysate concentrations were placed in duplicate wells of the 96-well plate of the FGF7 ELISA Kit (Cat. # ABIN6955870, Abbexa LLC, Sugar Land, TX, USA). FGF7 concentrations in each sample were derived from the average optical density (OD) using a graphed curve of OD versus FGF7 concentration for an FGF7 dilution series evaluated on the plate in duplicate. Final concentrations were corrected for dilutions of samples used.

### 2.10 FGF7 protein simple Jess assay

The 12- to 230-kDa Separation Module (Bio-Techne R&D Systems #SM-W001, Minneapolis, MN, USA) was used for electrophoresis separation of whole protein lysates in a capillary. Whole protein lysates were mixed with Simple Western Sample Buffer (0.1×) (Bio-Techne R&D Systems #PS-ST01EZ, Minneapolis, MN, USA) to a final concentration of 1.5 µg/µl and then denatured through heating at 96°C for 5 min. The FGF7 in the samples were detected using a primary antibody, Anti-KGF/FGF-7 antibody, which was validated by Bio-Techne for use on the ProteinSimple (ABCAM, EPR7261, Cambridge, UK), secondary antibody (Bio-Techne R&D Systems #DM-001, Minneapolis, MN, USA), and Chemiluminescent Substrate (Bio-Techne R&D Systems, Minneapolis, MN, USA), which were dispensed into designated wells in the Jess Simple Western plate. The plate was then centrifuged at 2,500 rpm for 5 min before being placed along with a capillary cartridge into the Protein Simple Jess instrument. The Compass program for Simple Western (Bio-Techne, Minneapolis, MN, USA) was used for initial analysis; peaks were chosen based on the molecular weight markers. Values of the area under the curve in the Compass Software were transferred to GraphPad Prism 10.2.1 for further analysis.

### 2.11 Analysis of public databases

KM-plotter (https://kmplot.com/) was used to analyze gene expression data (TCGA, GEO, EGA databases) for associations of FGF7 mRNA expression with survival probability of patients with ovarian cancer (25, 26).

### 2.12 Clinical specimens and data

Serum and frozen tumor specimens from patients with ovarian cancer who gave informed consent were obtained from the Stephenson Cancer Center Biospecimen Bank under IRB protocol #3260. Ascites specimens were collected from patients who consented to the IRB protocol #15066. Patient electronic medical records were searched under IRB protocol #73208 to determine the progression-free survival (PFS: months of time between date of diagnosis and date of recurrence or last follow-up) or overall survival (OS: months of time between date of diagnosis and date of death).

### 2.13 Statistical analysis

Differentially expressed genes were identified as described above in the NanoString analysis and Rosalind pipeline analysis sections. FGF7 protein levels were found to be normally distributed through the Shapiro–Wilk test, and groups were compared by either a paired or unpaired t-test. FGF7 protein levels in patient serum were categorized as above or below the median, and patient PFS or OS were compared between the two groups using the log-rank test. Patient demographics were compared using the Fisher’s exact test. Values of p or Q less than 0.05 were considered significant.

## 3 Results

### 3.1 Identification of differentially expressed genes in an orthotopic ovarian cancer model

**Figure 1:**
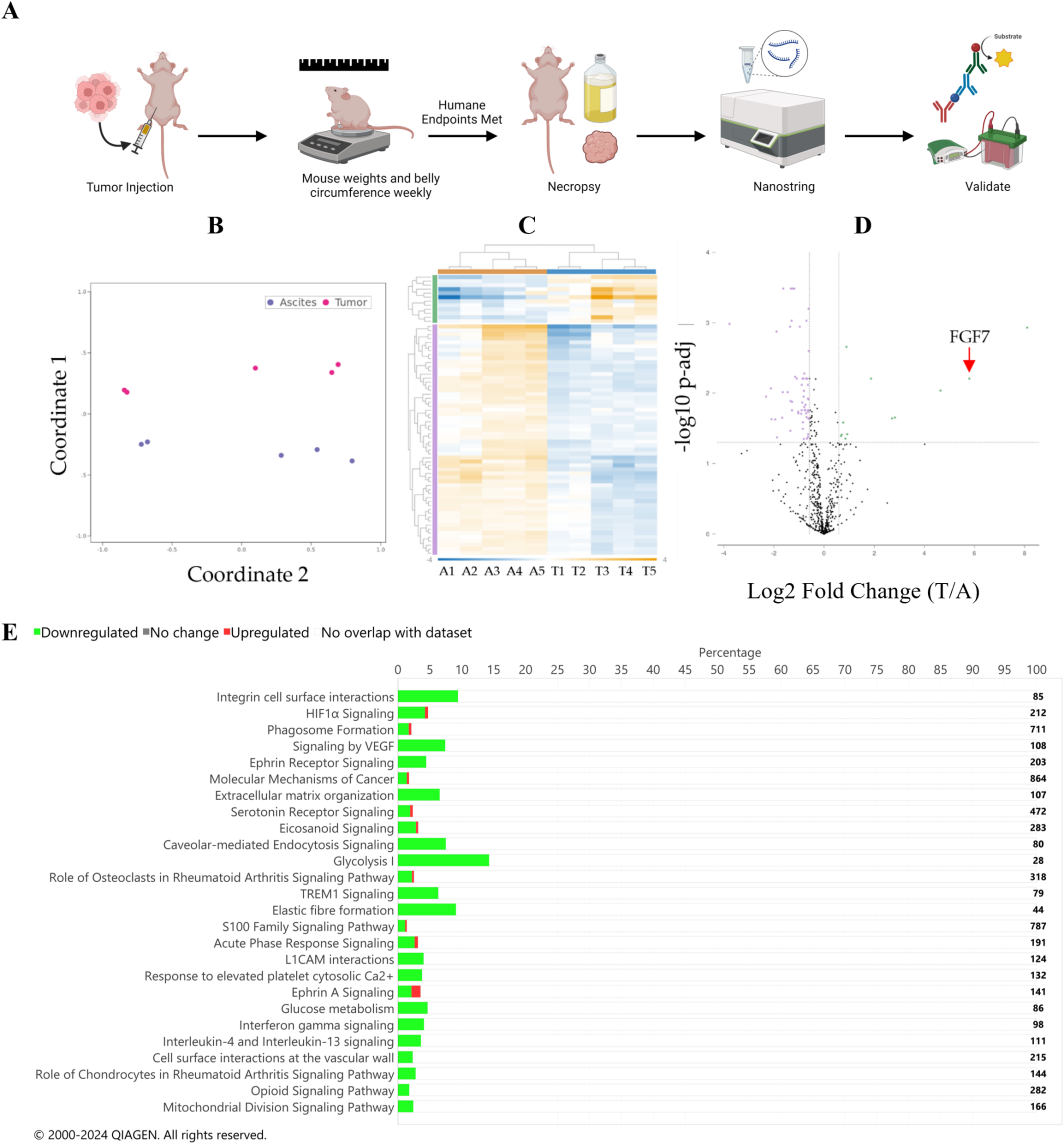
**(A)** Diagram of the study design used in the mouse models, which was generated using BioRender.com. Differential expression gene analysis. **(B)** Principal coordinate analysis plot. Purple represents ascites specimens, and pink represents tumor specimens. **(C)** Heat map of differential gene expression. Sample names are listed at the bottom of the map (A, ascites; T, tumor). Hierarchical clustering of samples is at the top of the map: orange represents ascites samples, and blue represents tumor samples. Fold change in the chart is depicted by color as indicated in the blue-to-orange scale bar at the bottom of the map and spans from −4 (blue) to 11 (orange). Gene hierarchical clustering is depicted on the left of the graph, green: upregulated in the tumor compared to the ascites, purple: downregulated in the tumor compared to the ascites. **(D)** Volcano plot of differential gene expression analysis. Green indicates genes that are upregulated in the tumor compared to those in the ascites. Purple indicates genes that are downregulated in the tumor compared to those in the ascites. The dotted horizontal line represents a Q value of 0.05, and the dotted vertical lines represent a Log2 fold change of 1.5 or −1.5. **(E)** IPA pathway result bar graph. Green is downregulated in the tumor compared to that in the ascites; red is upregulated in the tumor compared to that in the ascites. The number of total genes in each pathway is depicted to the right of the chart. Significance was defined by a Q value of <0.05 and a Log2 fold change of tumor divided by ascites >2.

The human ES-2 ovarian cancer cell line was used in an orthotopic model of ovarian cancer to generate tumor and ascites specimens for gene expression analysis (**Figure 1A**). We considered the peritoneal tumors established in this model to mimic metastatic lesions because they were generated from a cell line that was derived from a primary tumor. RNA was isolated from ascites cells or solid tumor specimens and evaluated for the expression of a panel of 770 cancer-related genes in the nCounter Tumor Signaling 360 Pathway Panel (**Supplementary Table S1**). Principal coordinates analysis (multidimensional scaling) demonstrated that the ascites cells and attached tumor samples clustered separately (**Figure 1B**). A differential gene analysis correcting for a false discovery rate of 5% identified a series of genes to be differentially expressed between the two specimen types (**Figure 1C**, **Supplementary Table S2**). Of those genes, FGF7 was one of the most differentially expressed (**Figure 1D**) with a Log fold change of −3.02 and a corrected p-value (Q value) of 0.024 (**Supplementary Table S2**). The complete list of normalized counts for all genes is provided in the **Supplementary Information** (**Supplementary Table S3**).

#### 3.1.1 Pathway analysis

**Figure 2:**
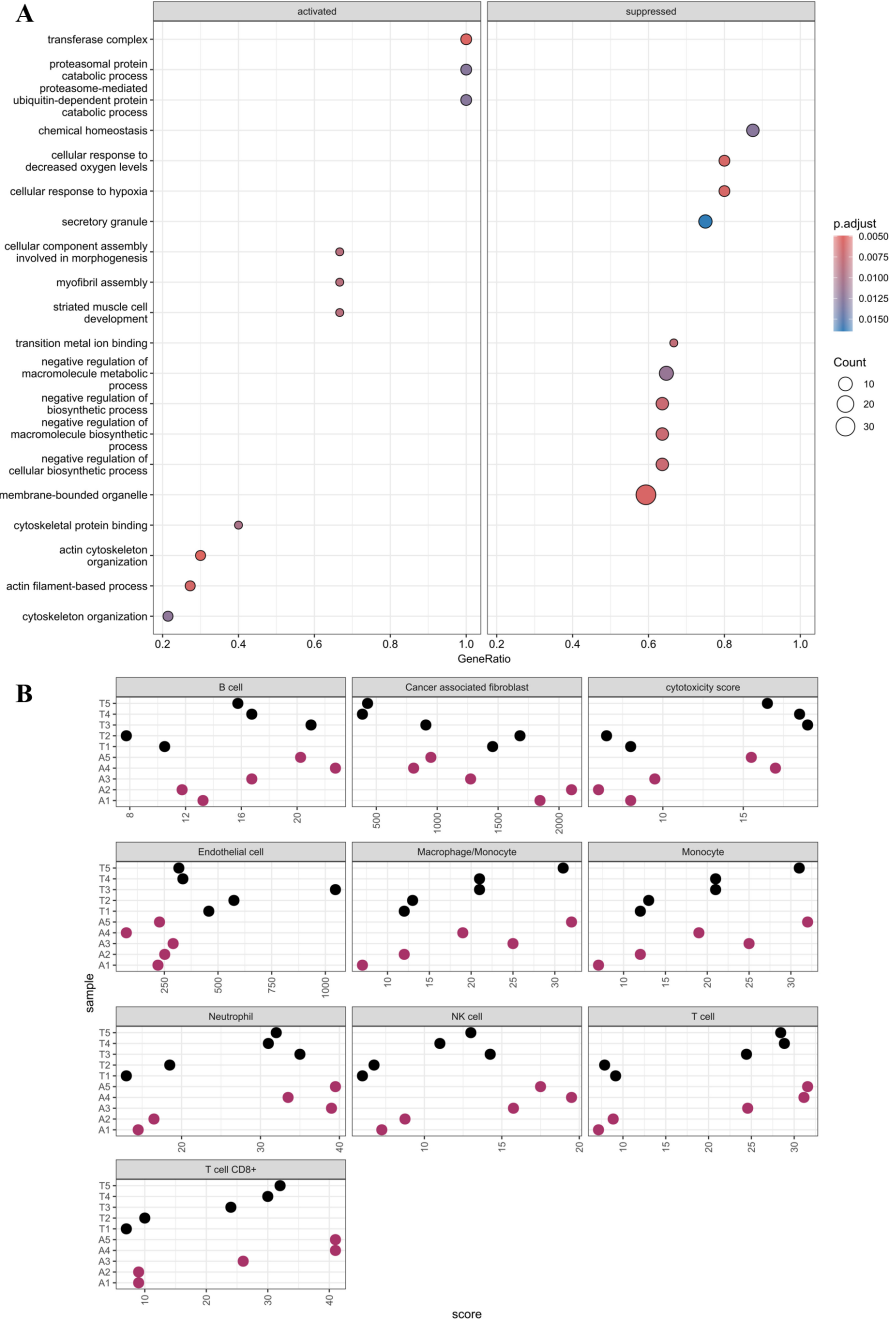
**(A)** GO pathways analysis. The chart is divided into activated and suppressed where the color and size of the dot indicate the p-adjusted value and count, respectively. **(B)** MCP counter plot. Cell type is labeled above each plot. Sample names are labeled along the y-axis and associated score on the x-axis.

Using IPA, 26 pathways were identified to be significantly different in the tumor compared to those in the ascites specimens (**Figure 1E**, **Supplementary Table S4**). Multiple inflammatory pathways were identified to be increased in ascites compared to those in the tumor specimens, while multiple other pathways were identified to be increased in the tumor compared to those in ascites specimens, such as integrin cell surface interactions, HIF1α signaling, phagosome formation, and signaling by VEGF. Additionally, a GO pathway analysis was performed and identified hypoxia and metabolic pathways, which is consistent with the IPA analysis (**Figure 2A**).

The data were further analyzed to predict the percentages of cancer, stromal, and immune cells in the specimens using the immunedeconv package in R. Tumor and ascites purity were estimated to be 85% (**Table 1**). Patterns of immune cell types and cytotoxicity scores were found to be similar, while the endothelial cells were higher, in the tumor and ascites specimens (**Figure 2B**). As expected, endothelial cells were significantly higher in the tumor compared to those in the ascites (**Table 2**). There was no statistical significance when comparing the other cell types.

**Table 1:** Estimated sample components.

	A1	T1	A2	T2	A3	T3	A4	T4	A5	T5
Stromal score	−179.11	−164.48	−176.41	−169.69	−166.61	−176.22	−172.88	−164.78	−173.76	−163.99
Immune score	−121.79	−123.60	−130.14	−125.78	−122.94	−130.17	−117.61	−124.44	−122.58	−132.54
ESTIMATE score	−300.89	−288.07	−306.54	−295.47	−289.55	−306.39	−290.49	−289.22	−296.34	−296.53
Purity	0.85	0.85	0.85	0.85	0.85	0.85	0.85	0.85	0.85	0.85

**Table 2:** p-Values of MCP plots comparing tumor and ascites scores.

	T cell	T-cell CD8+	Cytotoxicity score	NK cell	B cell	Monocyte	Macrophage/monocyte	Neutrophil	Endothelial cell	Cancer-associated fibroblast
p-Value^†^	0.841	0.659	0.452	0.222	0.460	0.873	0.873	0.548	0.008	0.310

^†^Mann–Whitney test (unpaired).

### 3.2 Validation of FGF7 protein differential expression in independent mouse models and patient samples

**Figure 3:**
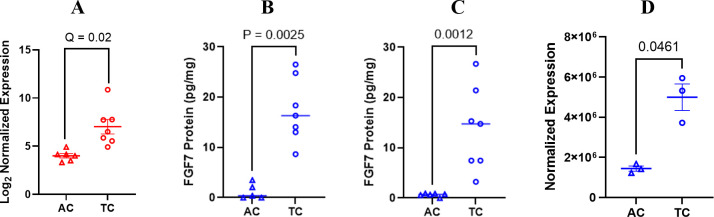
Differential expression of FGF7 gene and protein. **(A)** FGF7 RNA expression shown in Log2 normalized expression; false discovery rate of 5% was used in the analysis. **(B)** ELISA measurement of FGF7 protein in initial ES-2 orthotopic model of ovarian cancer compared using a paired t-test; **(C)** ELISA measurement of FGF7 protein in independent orthotopic ES-2 model of ovarian cancer compared using an unpaired t-test. **(D)** Capillary electrophoresis measurement of FGF7 in ID8 Trp 53^−/−^ model compared using an unpaired t-test. AC, ascites from the control group; TC, tumor from the control group.

**Figure 4:**
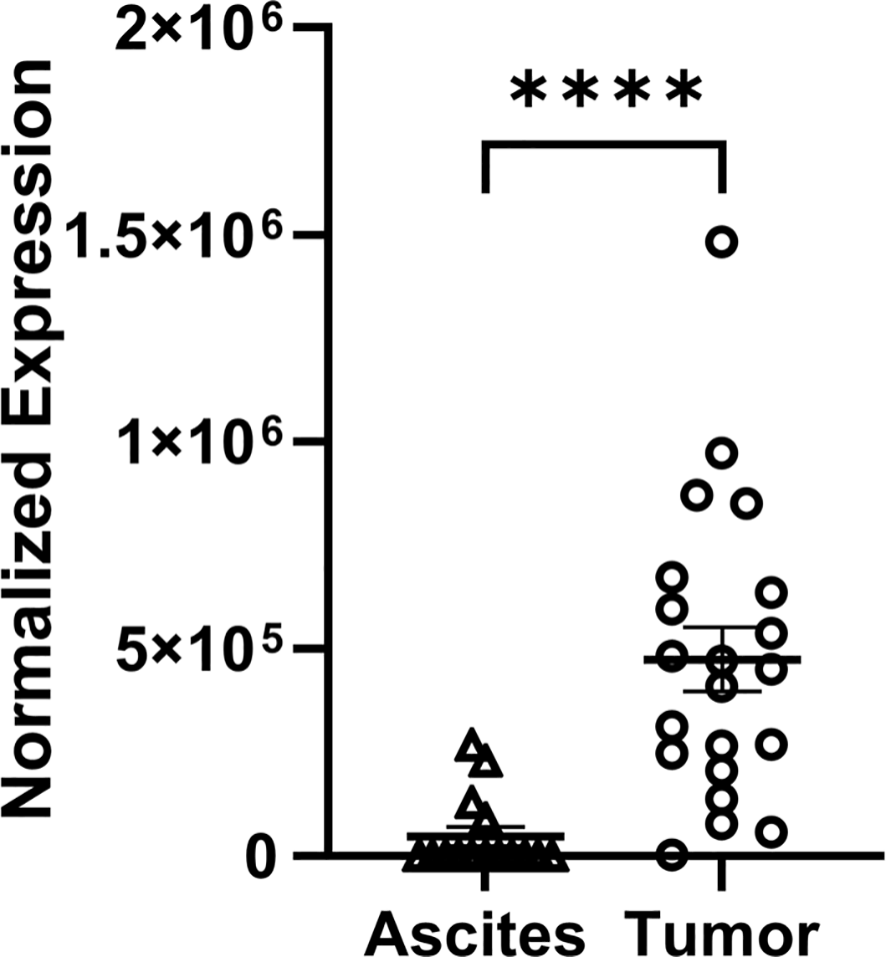
Differential expression of FGF7 protein in tumor and ascites samples collected from patients with ovarian cancer. Capillary electrophoresis measurement of FGF7 in human tumor and ascites samples compared using an unpaired t-test. ****p < 0.0001.

Differential expression of FGF7 at the protein level was validated using an ELISA to measure FGF7 in whole-cell protein extracts from mice treated in the discovery model used for the RNA analysis (**Figure 3A**) and from mice in a separate replicate mouse model using the same ES-2 cell line (**Figures 3B, C**). To further validate these findings, we conducted an additional experiment using an independent mouse model and different technologic assay for measuring FGF7. The independent mouse model utilized the ID8 *Trp 53^−/−^* luc syngeneic cell line in immunocompetent C56Bl/6 mice, while the independent technological approach involved a capillary nanoimmunoassay (**Figure 3D**). In all of these mouse models, FGF7 protein was expressed at significantly higher levels in the tumor compared to the ascites of ovarian cancer cell specimens. Our next validation step was to compare FGF7 protein levels in surgically collected tumor specimens with ascites specimens from patients with ovarian cancer. We considered the majority of the patient tumors to be metastatic lesions because the cells that formed the tumors originated at other organ sites in the patients, such as the fallopian tube and endometrium, before establishing at the ovary (27). The tumor samples expressed FGF7 at significantly higher levels when compared to the unmatched ascites samples (**Figure 4**, p < 0.0001).

### 3.3 Association of FGF7 with ovarian cancer patient survival

**Figure 5:**
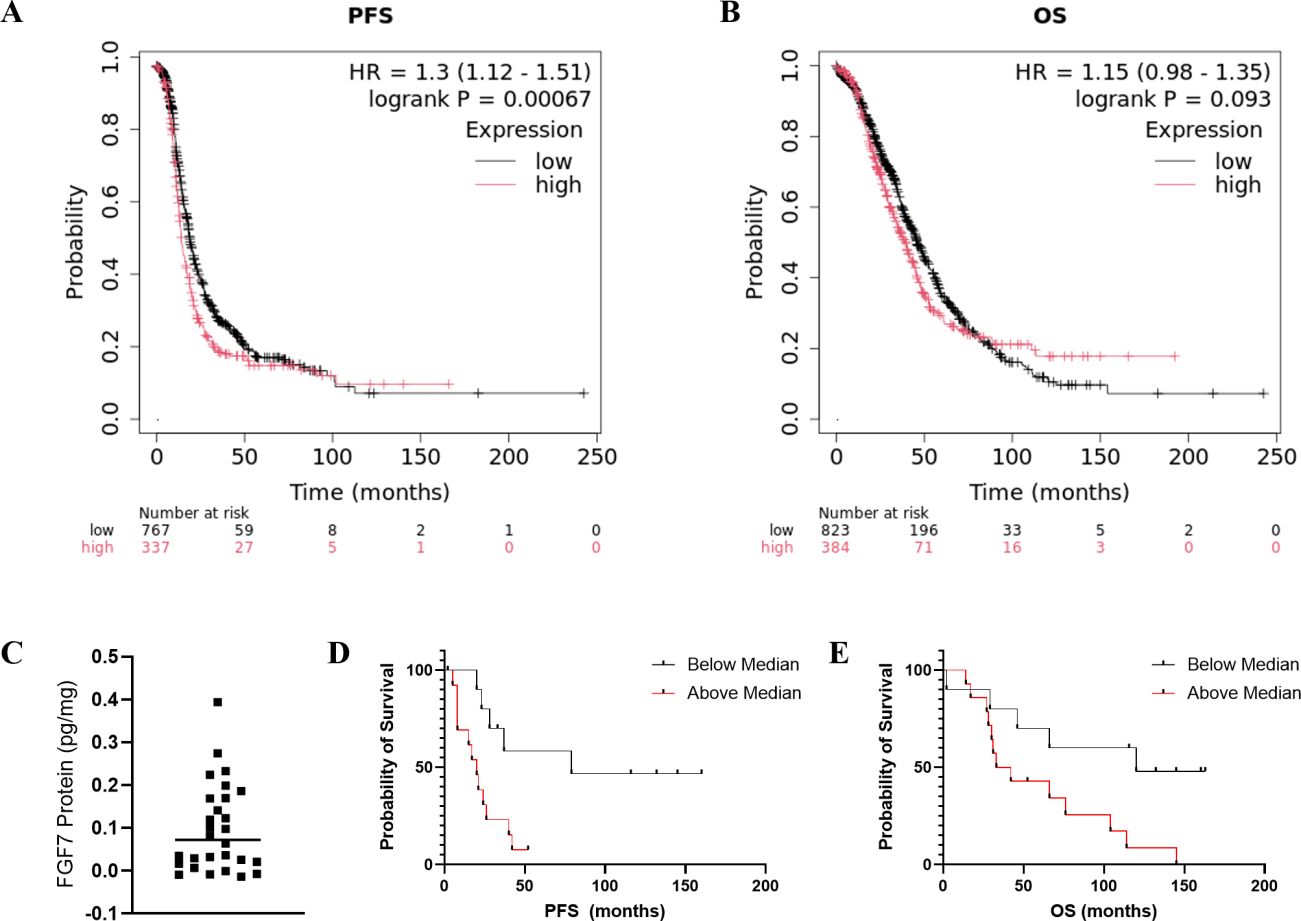
**(A, B)** Predictive value of tumor FGF7 protein expression. PFS and OS of ovarian cancer patients with high versus low tumoral FGF7 mRNA levels determined using KM-plotter. Serum FGF7 expression pattern and associations with patient survival. **(C)** Values of individual patient serum FGF7 levels. The horizontal line indicates the mean. **(D)** Comparison of PFS probability for patients with FGF7 above versus below the median. **(E)** Comparison of OS probability for patients with FGF7 above versus below the median. Statistical analysis: Log-rank test.

Analysis of the public databases revealed a significant association of higher *FGF7* gene expression with worse patient PFS probability (**Figure 5A**, p = 0.00067) and a trend of higher *FGF7* gene expression with worse OS (**Figure 5B**, p = 0.093). To evaluate whether serum FGF7 protein levels could be used to predict patient survival, we utilized 25 banked serum specimens from patients with ovarian cancer and a commercial FGF7 ELISA kit. The FGF7 protein exhibited a pattern of two groups with levels above versus below the median FGF7 protein value (**Figure 5C**). Comparison of patient groups with serum FGF7 levels above the median versus below the median demonstrated significant association of higher serum FGF7 protein levels with worse PFS for patients with ovarian cancer (Log-rank test p = 0.005, **Figure 5D**) and also with OS (Log-rank test p = 0.019, **Figure 5E**). Comparison of the demographics of the patients with low versus high FGF7 protein levels showed no significant differences in age, race, body mass index (BMI), tumor histology, cancer stage, blood cancer antigen 125 (CA-125), or exposure to bevacizumab in their treatment regimen (**Table 3**). This lack of significant differences in patient and tumor demographics indicates that the differences in survival between FGF7 low- and high-expressing groups are not due to any of these parameters.

**Table 3:** Comparison of patient demographics between FGF7 low versus high groups.

	FGF 7 low: N (%)	FGF 7 high: N (%)	p-Value^†^
Age	<65 years old: 3 (27) >65 years old: 8 (73)	<65 years old: 1 (8) >65 years old: 12 (92)	ns
Race	AI/AN: 1 (9) Asian: 0 Black: 1 (9) White: 9 (82)	AI/AN: 0 Asian: 1 (8) Black: 1 (8) White: 11 (84)	ns
BMI (kg/m^2^)	<18.5: 0 18.5–24.9: 2 (18) 25.0–29.9: 4 (36) >30.0: 5 (45)	<18.5: 0 18.5–24.9: 2 (15) 25.0–29.9: 6 (46) >30.0: 5 (38)	ns
Histology	Clear cell: 1 (9) Mucinous: 0 HGSOC: 9 (82) LGSOC: 1 (9)	Clear cell: 1 (8) Mucinous: 1 (8) HGSOC: 11 (84) LGSOC: 0	ns
Stage at diagnosis	I: 2 (18) II: 0 III: 7 (64) IV: 2 (18)	I: 1 (8) II: 0 III: 7 (54) IV: 5 (38)	ns
CA-125 at diagnosis	Normal (<35): 3 (27) Abnormal (>35): 8 (73)	Normal (<35): 2 (15) Abnormal (>35): 11 (84)	ns
Received bevacizumab	Yes: 6 (54) No: 4 (36) Unknown: 1 (9)	Yes: 6 (46) No: 6 (46) Unknown: 1 (8)	ns

HGSOC, high-grade serous ovarian cancer; LGSOC, low-grade serous ovarian cancer; AI/AN, American Indian/Alaskan Native; ns, not significant or >0.05; †Fisher’s exact test.

## 4 Discussion

In this study, we endeavored to identify and validate proteins responsible for the high rate of metastatic spread and recurrence in patients with ovarian cancer. We postulated that proteins differentially expressed in ovarian cancer cells present in solid tumors compared to peritoneal ascitic fluid play roles in the establishment of solid tumors from cancer cells present in ascites. Among the most differentially expressed genes identified in this study, FGF7 has known functions that could drive the establishment of metastatic ovarian lesions. The FGF7 protein is secreted by fibroblasts and induces growth, differentiation, and angiogenesis in epithelial cells and tumors (28) consistent with its alternate name of keratinocyte growth factor (KGF). Also, the expression patterns of FGF7 and its cell-surface receptors support targeting them in ovarian cancer drug development. FGF7 binds to the IIIb spliced version of the fibroblast growth factor receptor 2-IIIb (FGFR2-IIIb), which is exclusively expressed in epithelial cells. FGFR2-IIIb has been shown to be expressed in 80% of epithelial ovarian cancers, but not in normal ovarian surface epithelium (29). The FGF7 protein is expressed in 60% of ovarian cancers, as well as in normal ovarian surface epithelium. However, since normal ovarian surface epithelium does not express the IIIb isoform of FGFR2, it cannot respond to FGF7, and therefore, FGFR2-IIIb represents a rational cancer drug target (29, 30). Treatment of ovarian cancer cell lines with FGF7 increased invasion, while an antagonistic FGFR2-IIIb antibody reduced basal and FGF7-elevated invasion (31). Reduction of FGF7 and/or FGFR2 using shRNAi in ovarian cancer cell lines and tumors inhibited growth and increased cisplatin sensitivity (29). In cell culture, these findings were associated with a G2 cell cycle arrest and validated with a neutralizing FGF7 antibody. An allosteric inhibitor of FGFR2 IIIb and the alternatively spliced FGFR2 IIIc, alofanib, induced apoptosis in the SKOV3 ovarian cancer cell line and reduced growth of SKOV3 xenograft tumors in association with inhibition of angiogenesis (32).

Our study demonstrated that, in addition to FGF7 mRNA, the FGF7 protein was also significantly upregulated in the tumors compared to ascites in two replicates of the mouse model, a syngeneic immunocompetent mouse model and clinical specimens collected from patients with ovarian cancer. Furthermore, we demonstrated that higher FGF7 levels in serum were associated with worse ovarian cancer patient outcome. A machine learning study of RNA expression profiles found prognostic significance of elevated FGF7 mRNA with worse ovarian cancer patient survival probability (28); however, to date, there are no reports of FGF7 protein prognostic significance in patients with cancer. Our study contributes new information that FGF7 protein also has prognostic significance for patients with ovarian cancer. Furthermore, serum was used as the specimen type analyzed in this study. Use of serum in clinical diagnosis represents a less-invasive means to evaluate patient prognosis and monitor response to treatment.

It is possible that our observation of lower expression of FGF7 in ascites compared to tumor cells is due to reduced percentages of cancer-associated fibroblasts in the ascites specimens in comparison to the adherent tumor. However, our bioinformatic analysis of the transcriptomic data estimated that there were no significant differences in the percentage of cancer-associated fibroblasts in the tumors compared to those in the ascites. There were also no significant differences in immune cell profiles between the two specimen types, suggesting that immune-based therapy could be equally effective against solid tumors and floating ascites cells or spheroids.

One interpretation of our observation of higher FGF7 in tumors is that once the ovarian cancer cells invaded into tissue, those cells, which were able to establish as viable tumors, needed high FGF7 expression to stimulate angiogenesis to support tumor growth. This is consistent with the significantly higher percentages of endothelial cells in the tumor compared to those in the ascites. Solid tumors require development of blood vessels to grow beyond 1–2 mm (33), whereas ascites spheroids are able to survive and replicate without the development of blood vessels.

Several FGFR inhibitors are FDA approved for use in non-gynecologic malignancies. Pemigatinib (FGFR1-3 inhibitor) and futibatinib (FGFR1-4 inhibitor) are approved for previously treated, unresectable, locally advanced or metastatic intrahepatic cholangiocarcinoma with FGFR2 fusions or other rearrangements. Pemigatinib is also approved for relapsed or refractory myeloid/lymphoid neoplasms with FGFR1 gene rearrangement (34). Bemarituzumab (humanized monoclonal antibody against FGFR2-IIIb) is approved for FGFR2-IIIb-overexpressing and HER2-negative metastatic and locally advanced gastric and gastroesophageal adenocarcinoma in combination with modified FOLFOX6 (fluoropyrimidine, leucovorin, and oxaliplatin) based on the FIGHT trial (35). Finally, erdafitinib (FGFR1-4 inhibitor) is approved for locally advanced or metastatic urothelial carcinoma with susceptible FGFR3 genetic alterations based on a phase III randomized controlled trial (36).

In addition to this potential for developing ovarian cancer therapies targeted at FGF7/FGFR2-IIIb, FGF7 has potential for being used as a biomarker of patient prognosis and sensitivity to FGFR inhibitors. Analysis of public databases identified associations of high *FGF7* gene expression with worse ovarian cancer patient survival probability. Our analysis of serum FGF7 protein levels from patients with ovarian cancer found FGF7 protein expression above the median to be associated with worse progression-free survival and overall survival independent of patient and tumor characteristics. The use of serum as a surrogate for the tumor offers a less-invasive opportunity to detect and monitor biomarkers of prognosis, treatment response, and recurrence.

While our study provides proof-of-principal that our approach can identify candidate drug target and predictive biomarkers, the other differentially expressed genes could also be evaluated one-by-one or using a bioinformatic approach. Analysis of the pathways predicted by the differentially expressed genes provides information of the biological differences between tumor and ascites, which could be used to better understand the process by which ascites cells attach, invade, and grow as tumors. In our study, the attached tumor cells exhibited increased percentages of endothelial cells and decreased hypoxia reflective of tumor angiogenesis providing a blood supply of oxygen in attached tumors, and not in aggregates or spheroids of cancer cells in ascitic fluid. Also, the tumors exhibited increased proteasomal catabolism and macromolecule metabolism in comparison to ascitic fluid cells, suggesting that the tumors utilize recycling as a survival mechanism to compensate for the reduced amount of nutrients available within tissue in comparison to ascitic fluid. The increased processes involving cytoskeletal organization in the tumor compared to ascites cells are consistent with increased capacity of the cells to attach, invade, and migrate. Further study of these specific differentially expressed genes and pathways has the potential to identify biomarkers of metastases and drug targets for inhibiting metastases.

A limitation of our study is the use of cell lines with indeterminant ovarian cancer histologies. Ovarian cancer is a highly heterogeneous disease with multiple distinct histologic types that arise from different organ sites and exhibit unique DNA mutation profiles (27). The histologic types include HGSOC, low-grade serous, clear cell, mucinous and endometrioid, with the HGSOC histology being the most common and lethal (27, 37). The human ES-2 ovarian cancer cell line used in this study was derived from a clear cell carcinoma; however, its genetic and protein expression patterns exhibit characteristics of HGSOC (38, 39). The murine ID8 *Trp53^−/−^* cell line used in this study was generated from the parental ID8 cell line by CRISPR/Cas9-mediated knockout of the *Trp53* gene (18). While the ID8 cells exhibit characteristics of clear cell carcinoma, the genetically altered ID8 *Trp53* ^−/−^ cell line exhibits characteristics of HGSOC. Our evaluation of human clinical specimens from patients with multiple different ovarian cancer histologies does not provide sufficient numbers to make conclusions based on histologic type, which will be an important aspect of future research. The pathways identified to be upregulated in the tumor compared to the ascites of our ES-2 model by IPA analysis of differentially expressed gene patterns are consistent with processes known to be involved in the establishment of ovarian cancer metastases: integrin–cell surface interactions, signaling by VEGF, molecular mechanisms of cancer, and extracellular matrix reorganization pathways (40). This observed tumoral upregulation of pathways known to be involved in ovarian cancer metastasis supports the validity of our model to identify candidate ovarian cancer biomarker and drug targets. The observed upregulation of multiple inflammatory pathways in the ascites compared to the tumor specimens is consistent with the large number of immune cells in ascites.

In conclusion, our study identified a clinically significant transcriptomic difference between tumor and ascites cells and provided proof-of-principal that studying differentially expressed genes between tumor and ascites cells can identify candidate cancer drug targets and predictive biomarkers. The results also identify candidate mechanisms of the process of ovarian cancer metastases and recurrence. Overall, the data presented reveal that FGF7 mRNA and protein are significantly overexpressed in solid tumors compared to ascites cells supporting the utility of FGF7 as a rational target for pharmacological intervention in solid tumors. Preclinical data justifies targeting the FGF7/FGFR2 pathway in ovarian cancer. Published studies of others document that genetic and pharmacologic inhibition of FGF7 and/or FGFR2 inhibits ovarian cancer tumor growth. While no anti-FGF7 drugs are currently in clinical trial, several anti-FGFR therapies have proven effective in phases I–III trials for various cancer types leading to multiple FDA approvals. Based on knowledge of the FGF7/FGFR2-IIIb interaction, bemarituzumab, which specifically targets FGF7’s receptor FGFR2b, would be of particular interest and may serve as a promising agent for future use in ovarian cancer. Furthermore, FGF7 protein is a rational candidate biomarker for prediction of ovarian cancer patient prognosis.

## Data Availability

The original contributions presented in the study are included in the article/supplementary material, further inquiries can be directed to the corresponding author.
